# Modality-Specificity of the Neural Correlates of Linguistic and Non-Linguistic Demand

**DOI:** 10.1162/nol_a_00114

**Published:** 2023-09-18

**Authors:** Mackenzie Philips, Sarah M. Schneck, Deborah F. Levy, Stephen M. Wilson

**Affiliations:** Department of Hearing and Speech Sciences, Vanderbilt University Medical Center, Nashville, TN, USA; School of Health and Rehabilitation Sciences, University of Queensland, Brisbane, Australia

**Keywords:** accuracy, aphasia, linguistic demand, multiple demand network, reaction time, task difficulty

## Abstract

Imaging studies of language processing in clinical populations can be complicated to interpret for several reasons, one being the difficulty of matching the effortfulness of processing across individuals or tasks. To better understand how effortful linguistic processing is reflected in functional activity, we investigated the neural correlates of task difficulty in linguistic and non-linguistic contexts in the auditory modality and then compared our findings to a recent analogous experiment in the visual modality in a different cohort. Nineteen neurologically normal individuals were scanned with fMRI as they performed a linguistic task (semantic matching) and a non-linguistic task (melodic matching), each with two levels of difficulty. We found that left hemisphere frontal and temporal language regions, as well as the right inferior frontal gyrus, were modulated by linguistic demand and not by non-linguistic demand. This was broadly similar to what was previously observed in the visual modality. In contrast, the multiple demand (MD) network, a set of brain regions thought to support cognitive flexibility in many contexts, was modulated neither by linguistic demand nor by non-linguistic demand in the auditory modality. This finding was in striking contradistinction to what was previously observed in the visual modality, where the MD network was robustly modulated by both linguistic and non-linguistic demand. Our findings suggest that while the language network is modulated by linguistic demand irrespective of modality, modulation of the MD network by linguistic demand is not inherent to linguistic processing, but rather depends on specific task factors.

## INTRODUCTION

This study is concerned with the question of how the brain responds to task difficulty in linguistic and non-linguistic contexts, and specifically, whether the modality in which stimuli are presented—auditory or visual—makes a difference. This question is relevant to the interpretation of functional imaging studies of language processing in post-stroke aphasia and other clinical populations, since processing effort can often be difficult to match or control in these types of studies (Brownsett et al., 2014; Fridriksson & Morrow, 2005; Price et al., 2006; Raboyeau et al., 2008; Sharp et al., 2004, 2010; Wilson et al., 2018, 2019) and often confounds comparisons between patients and controls, correlations among patients varying in severity, and longitudinal analyses of individuals who are changing over time (Geranmayeh et al., 2014; Quillen et al., 2021; Wilson & Schneck, 2021).

Several studies have compared how neural activity is modulated by linguistic and non-linguistic demand (Eckert et al., 2009; Erb et al., 2013; Piai et al., 2013; Quillen et al., 2021). The present study is a follow-up to the recent study by Quillen et al. (2021), which is, to our knowledge, the first study to directly compare the brain regions modulated by linguistic demand and non-linguistic demand by closely matching task structure and behavioral data across the linguistic and non-linguistic domains. In Quillen et al. (2021), linguistic demand was operationalized by contrasting a difficult semantic matching task to an easier version of the same task, while non-linguistic demand was operationalized by contrasting difficult and easy variants of a perceptual judgment task. It was found that linguistic demand modulated not only left hemisphere language regions, but also a set of bilateral brain regions that have been termed the multiple demand (MD) network—the inferior frontal junction, anterior insula, pre-supplementary motor area (SMA), anterior-mid cingulate, and intraparietal sulcus. The MD network has been argued to support cognitive flexibility in many contexts (Duncan & Owen, 2000; Fedorenko et al., 2013). In contrast, non-linguistic demand did not modulate language regions, but modulated the MD network to an even greater extent. It was concluded that linguistic and non-linguistic demand have distinct neural correlates, which need to be carefully considered when interpreting clinical studies in which task performance varies across patients or groups.

Quillen et al.’s (2021) study was carried out in the visual modality: Participants made judgments about pairs of written words, or pairs of symbol strings. However, language processing of course takes place in other modalities too, most commonly the auditory modality. In the present study, we wanted to determine whether the neural correlates of linguistic demand (and non-linguistic demand) depend on the modality in which stimuli are presented. To determine whether the neural correlates of linguistic demand (and non-linguistic demand) depend on the modality of presentation, we carried out a parallel version of the Quillen et al. (2021) experiment, in which we presented the semantic matching task in the auditory modality rather than the visual modality, and we replaced the visual perceptual matching task with a structurally similar melodic matching task in the auditory modality.

We hypothesized that stimulus modality would have little effect, because difficulty manipulations should modulate higher level processes, not sensory processes. Previous studies involving contrasts that differed in terms of linguistic demand in the auditory modality have reported modulation of language regions (Lopes et al., 2016; Obleser et al., 2011; Rodd et al., 2005; Shain et al., 2020, 2022; Wehbe et al., 2021; Wilson et al., 2016) as well as likely MD regions (Binder et al., 2004; Davis & Johnsrude, 2003; Eckert et al., 2009; Erb et al., 2013; Lopes et al., 2016; Peelle, 2018; Vaden et al., 2013), similar to what Quillen et al. (2021) observed in the visual modality. Regarding non-linguistic demand, a recent study by Assem et al. (2022) reported very similar patterns of activation for contrasts between hard and easy *n*-back tasks presented in the auditory modality or the visual modality: The difficulty contrast robustly activated the MD network in both cases. On the other hand, there are reasons to question whether aspects of Quillen et al.’s (2021) findings might reflect the visual modality of presentation. Several parts of the MD network appear to have visual functions: the dorsal premotor and intraparietal sulcus nodes are involved in spatial attention and eye movements (Corbetta & Shulman, 2002, 2011), and there is an occipito-temporal MD node (Fedorenko et al., 2013) that may potentially reflect attentional modulation of visual representations (Duncan, 2013). All these regions were modulated by linguistic demand in Quillen et al. (2021), so we wanted to determine whether that would still be the case when spatial attention, eye movements, and visual representations were removed from the equation.

## MATERIALS AND METHODS

### Participants

Nineteen neurologically normal individuals (mean age 26.9 ± 3.9 (*SD*) yr, range 22–38 yr; 2 male, 17 female; 17 right-handed, 1 left-handed, 1 ambidextrous; 18 native speakers of English, 1 fluent in English; education 17.7 ± 1.2 yr, range 16–20 yr) were successfully scanned with functional magnetic resonance imaging (fMRI). Participants were recruited by word of mouth from Vanderbilt University Medical Center. All participants were left-lateralized for language, as revealed by contrasts of semantic and melodic conditions; an additional two participants (both left-handed) were scanned but excluded as they showed clear evidence of right hemisphere dominance for language.

All participants gave written informed consent and were compensated for their time. The study was approved by the institutional review board at Vanderbilt University Medical Center.

### Experimental Design

**Figure 1.**
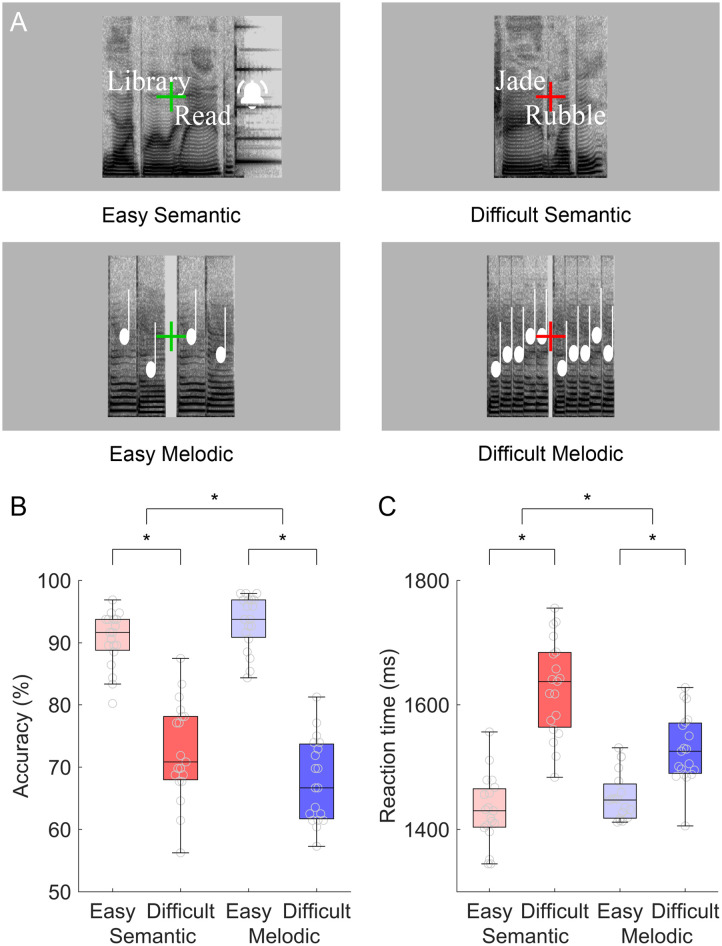
Experimental design and behavioral data. (A) Example items from the four conditions. Spectrograms are shown for the auditory stimuli, and the words or tones are shown in white. The Semantic Easy item is a match, and the “ding” sound reflecting a button press for a match is shown. The actual visual display consisted solely of green (easy) or red (difficult) crosshairs on a gray background. (B) Accuracy by condition. (C) Reaction time by condition.

The fMRI design was closely modeled on the experiment described by Quillen et al. (2021), except that stimuli were presented in the auditory modality. There were five conditions in a block design: (1) Semantic Easy; (2) Semantic Difficult; (3) Melodic Easy; (4) Melodic Difficult; (5) Rest (Figure 1A). All blocks were 16 s in duration, and each block (except for the rest condition) included eight stimuli, which were presented every 2 s. A run consisted of six blocks per condition in pseudorandom order, for a total of 30 blocks, that is, exactly 8 min. Each participant was first trained on the task, then they performed one complete practice run prior to entering the scanner, so that they would be familiarized with the four active conditions and would settle on strategies for each condition. Finally, they performed two runs in the scanner while functional images were acquired, so there were 12 blocks per condition in all, containing a total of 96 trials per condition.

In the four active conditions, each trial consisted of a pair of words or a pair of melodies presented one after the other. Stimuli across the four conditions were matched for mean stimulus duration (Table 1) and root mean squared power. Participants were instructed to press a button with a finger of their left hand if the words “go together” or if the melodies “are identical,” and to do nothing otherwise. The response window began at the end of the first member of the pair, and extended to the end of the first member of the pair on the following trial, but reaction times were measured from trial onset. When the response button was pressed, a “ding” sound of duration 461 ms was played to acknowledge the button press, but no feedback was provided as to whether the response was correct.

**Table 1.** Characteristics of the stimuli.

**Condition**	**Duration (ms)**	**Frequency**	**Age of acquisition**	**Concreteness**	**Match example**	**Mismatch example**
Semantic Easy	1,234 ± 132	7.51 ± 0.89	4.62 ± 0.88	558 ± 60	RABBIT	TOMATO
					CARROT	BEACH
Semantic Difficult	1,251 ± 161	4.50 ± 0.89	9.63 ± 1.00	438 ± 87	SOAR	WHIFF
					FLUTTER	OUTCOME
Melodic Easy	1,224 ± 0	–	–	–	C4-E4	D4-E4
					C4-E4	D4-D4
Melodic Difficult	1,225 ± 0	–	–	–	C4-D4-C4-D4-E4	D4-C4-E4-C4-E4
					C4-D4-C4-D4-E4	D4-C4-E4-D4-E4

*Note*. Frequency is the average log lemma frequency across each pair; age of acquisition is the average across each pair in years; and concreteness is the average rating across each pair; see Quillen et al. (2021) for further details.

In the Semantic Easy condition (Table 1), half of the word pairs were semantically related, and half were not. The words were relatively high frequency, concrete, and acquired early, and the semantic relationships between the matching word pairs varied in their nature (e.g., synonyms, antonyms, associates, part-whole) but were chosen to be relatively transparent (see Quillen et al., 2021, and Wilson et al., 2018, for details). Words were recorded by a female speaker on a Marantz PMD661MKII digital voice recorder in a soundproof booth. In order to fit each trial into its planned 2 s presentation window, any words longer than 900 ms were reduced in length by 5% using the MATLAB function stretchAudio. The word pairs were presented sequentially but with the end of the first word overlapping the beginning of the second word by 200 ms. This was necessary to match stimulus duration across conditions. A green crosshair was displayed throughout each block so that participants knew when they were performing the easy condition.

The Semantic Difficult condition (Table 1) was the same as the Semantic Easy condition except that the words were relatively low frequency, abstract, and acquired later, and the semantic relationships between the matching word pairs were chosen to be relatively opaque (see Quillen et al., 2021, and Wilson et al., 2018, for details). Words were recorded, edited, and presented as just described, except that pairs overlapped each other by 250 ms in this condition. A red crosshair was displayed throughout each block so that participants knew when they were performing the difficult condition. Unlike in Quillen et al. (2021), to simplify the present experiment, the same words were presented to each participant, with no adaptation based on participant performance.

In the Melodic Easy condition (Table 1), each melody consisted of two notes. Each note was a sung syllable “da” of duration 276 ms at one of three pitches (C4, D4, or E4). There was a gap of 120 ms between the two melodies. Mismatching melodies were created by shifting one note by one tone. A green crosshair was displayed throughout each block.

The Melodic Difficult condition (Table 1) was the same as the Melodic Easy condition, except that each melody consisted of five notes, each with a duration of 117 ms, and there was a gap of 50 ms between the two melodies. Mismatching melodies were created by shifting one note by one tone. A red crosshair was displayed throughout each block.

### Neuroimaging

Participants were scanned on a Philips Achieva 3 Tesla scanner with a 32-channel head coil at the Vanderbilt University Institute of Imaging Science. Auditory stimuli were presented over MRI-compatible headphones (NordicNeuroLab) at a comfortable volume for each participant, which was determined by playing example stimuli over scanner noise prior to acquiring real data. Visual stimuli were projected onto a screen at the end of the bore, which participants viewed through a mirror mounted to the head coil. T2*-weighted blood oxygen level dependent echo planar images were collected with the following parameters: 240 volumes + 4 initial volumes discarded; 35 axial slices in interleaved order; slice thickness = 3.0 mm with 0.5 mm gap; field of view = 220 × 220 mm; matrix = 96 × 96; repetition time (TR) = 2,000 ms; echo time (TE) = 30 ms; flip angle = 75°; SENSE factor = 2; voxel size = 2.3 × 2.3 × 3.5 mm. T1-weighted structural images (voxel size = 1.0 × 0.8 × 0.8 mm) and coplanar T2-weighted images (voxel size = 0.4 × 0.4 × 3.5 mm) were also acquired.

### Behavioral Data Analysis

The behavioral data were analyzed with repeated measures analyses of variance (ANOVAs) in JMP Version 12.0.1 (SAS Institute). Accuracy was computed based on all trials (hits and correct rejections, versus misses and false alarms). Reaction times from all trials with button presses (i.e., hits and false alarms) were included in the analyses. To reduce the influence of outlier reaction times, within each condition for each participant, reaction times that were more than 2.5 standard deviations from the mean for that condition and that participant were set to 2.5 standard deviations from the mean; the number of trials clipped in this manner ranged from 1 to 8 per participant.

### Neuroimaging Data Analysis

The functional imaging data were analyzed using exactly the same procedure described by Quillen et al. (2021). In brief, we used AFNI and FSL for preprocessing, FMRISTAT for model fitting, and SPM for coregistration and intersubject normalization. *Linguistic demand* was modeled with the contrast Semantic Difficult − Semantic Easy. *Non-linguistic demand* was modeled with the contrast Melodic Difficult − Melodic Easy. The interaction of domain by difficulty was modeled by the contrast (Semantic Difficult − Semantic Easy) − (Melodic Difficult − Melodic Easy). The language network was identified with the contrast (Semantic Easy + Semantic Difficult) − (Melodic Easy + Melodic Difficult). Second level random effects analyses were performed and a cluster-defining threshold of *p* < 0.005 was applied. Correction for multiple comparisons was carried out by permutation testing of the maximum cluster extent using the FSL function “randomise” with 10,000 permutations.

To statistically examine the effect of modality (auditory, visual), the 19 participants in the present study were compared to the 20 participants who completed the analogous experiment in the visual modality in Quillen et al. (2021). Whole brain between-groups analyses included covariates of age, sex, handedness, and education, since these were not exactly matched between the two cohorts.

A region of interest (ROI) analysis was carried out to examine responses to each of the four active conditions in the language network (including several homotopic nodes in the right hemisphere) and the MD network, using ROIs that were functionally defined in individual participants (Fedorenko et al., 2010). The search regions for the language ROIs were spheres of 8 mm radius centered on peaks of the language contrast in Quillen et al. (2021); coordinates are provided in Supplementary Table 1 of the Supporting Information, available at https://doi.org/10.1162/nol_a_00114. For each of the two runs, we defined individual ROIs as the top 10% of voxels within each sphere that had the highest *t* statistics for the language contrast in the other run, and that were not modulated by non-linguistic demand (uncorrected *p* > 0.1). The search regions for the MD ROIs were also spheres of 8 mm radius centered on peaks of the MD contrast from Fedorenko et al. (2013); coordinates are provided in Supplementary Table 1. For each of the two runs, we defined individual ROIs as the top 10% of voxels within each sphere that had the highest *t* statistics for modulation by non-linguistic demand in the other run, and that were not modulated by language (uncorrected *p* > 0.1). Comparisons within participants and between participant groups were performed with *t* tests in MATLAB and were corrected for multiple comparisons using permutation testing. Specifically, for each set of ROIs (language, MD) and contrast of interest (linguistic demand, non-linguistic demand, interaction), we compared observed *t* statistics to null distributions of the maximal *t* statistic recorded across the set of regions under 10,000 permutations with sign flipping (within group analyses) or reassignment of group labels (between groups analyses).

## RESULTS

### Behavioral Data

For accuracy (Figure 1B), a repeated measures ANOVA with two within-subjects factors (domain, difficulty) revealed a main effect of difficulty with difficult conditions less accurate than easy conditions, *F*(1, 18) = 940.11, *p* < 0.0001, but no main effect of domain, *F*(1, 18) = 0.35, *p* = 0.56. Accuracy was reduced on difficult items both for the semantic conditions, 72.6 ± 7.9% compared to 90.5 ± 4.4%, *t*(18) = −12.09, *p* < 0.0001 and for the melodic conditions, 67.9 ± 6.7% compared to 93.4 ± 4.3%, *t*(18) = −16.56, *p* < 0.0001, confirming the success of the difficulty manipulations. There was an interaction of domain by difficulty, *F*(1, 18) = 8.03, *p* = 0.011, such that there was a larger effect of difficulty in the melodic conditions.

For reaction time (Figure 1C), a repeated measures ANOVA revealed a main effect of difficulty, with slower responses to difficult conditions compared to easy conditions, *F*(1, 18) = 378.17, *p* < 0.0001, and a main effect of domain, with slower responses in the semantic conditions, *F*(1, 18) = 12.25, *p* = 0.0026. Reaction times were slower on difficult items both for the semantic conditions, 1,628 ± 78 compared to 1,431 ± 55 ms, *t*(18) = 13.93, *p* < 0.0001 and for the melodic conditions, 1,529 ± 55 compared to 1,439 ± 72 ms, *t*(18) = 5.57, *p* < .0001, further supporting the success of the difficulty manipulations. There was an interaction of domain by difficulty, *F*(1, 18) = 16.43, *p* = 0.0007, such that there was a larger effect of difficulty in the semantic conditions.

The significant interactions of domain by difficulty for both accuracy and reaction time were not desired. However, the fact that these two interactions went in opposite directions means that there was an effect of difficulty in both the semantic and the melodic conditions, but these effects played out more in reaction time for the former and accuracy for the latter.

### Brain Regions Modulated by Linguistic Demand

**Figure 2.**
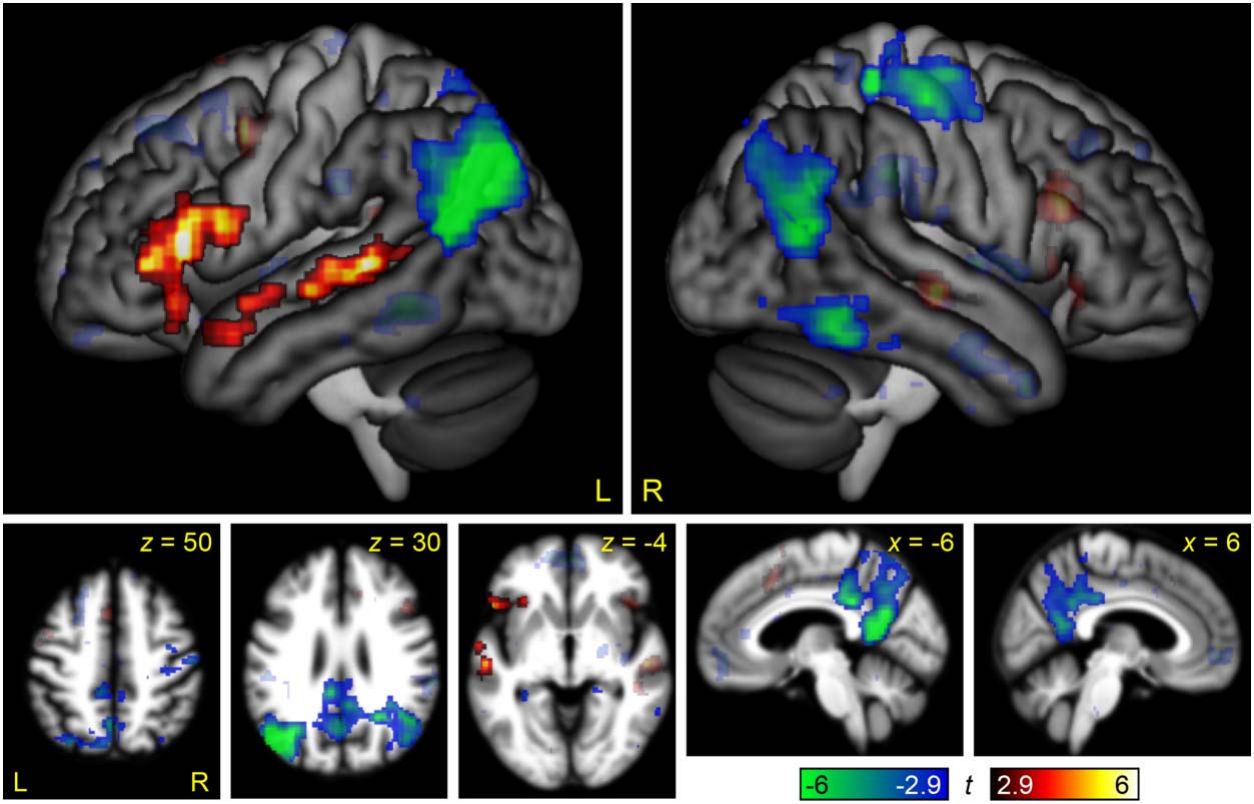
Brain regions modulated by linguistic demand. The contrast between the Semantic Difficult and Semantic Easy conditions is shown in hot colors, while the reverse contrast is shown in cool colors. Opaque = statistically significant, corrected for multiple comparisons; transparent = voxelwise *p* < 0.005, but did not meet cluster extent threshold.

**Figure 3.**
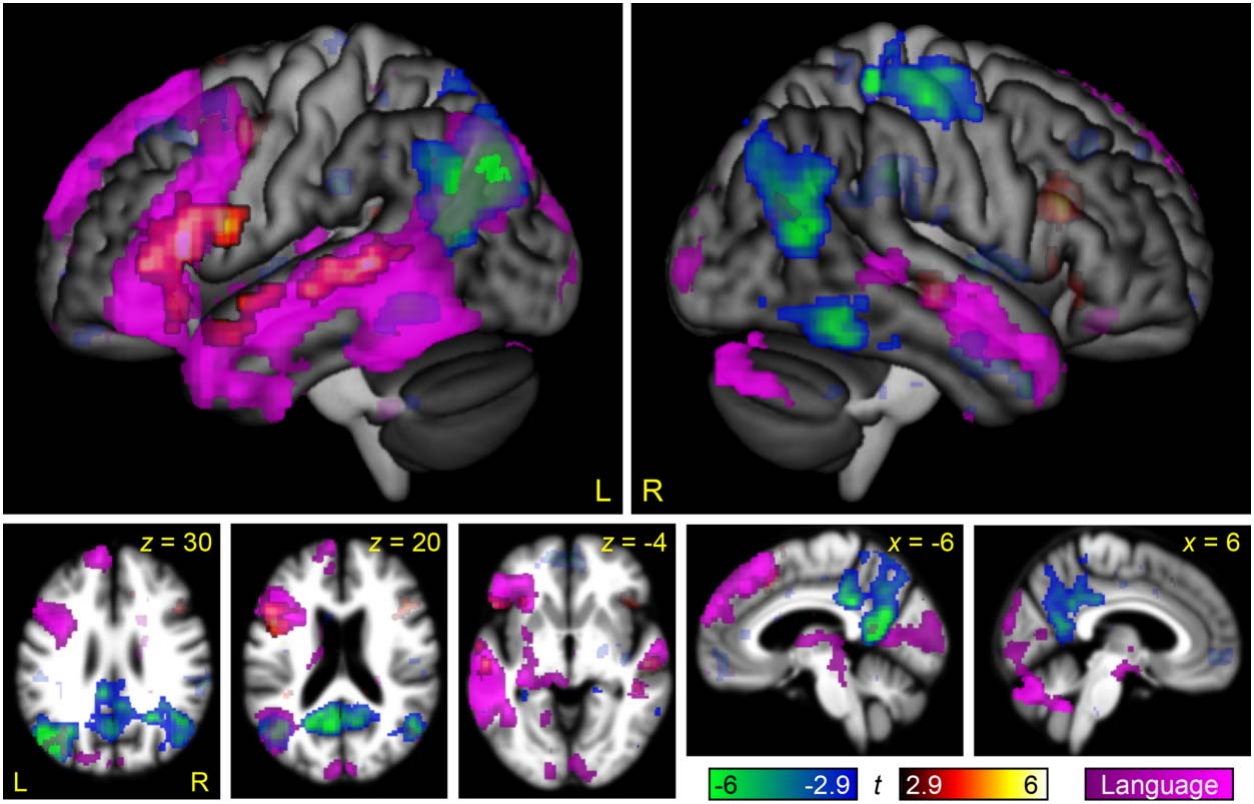
Brain regions modulated by linguistic demand in relation to language areas of the brain. Language areas, as revealed by the contrast of (Semantic Easy + Semantic Difficult) − (Melodic Easy + Melodic Difficult) are shown in purple, overlaid on the same linguistic demand activations shown in Figure 2. Opaque = statistically significant, corrected for multiple comparisons; transparent = voxelwise *p* < 0.005, but did not meet cluster extent threshold.

The contrast between the Semantic Difficult and Semantic Easy conditions was used to identify brain regions modulated by linguistic demand in the auditory modality (Figure 2 and Table 2). The regions that were differentially active for the more difficult condition were the left inferior frontal gyrus (IFG; primarily pars opercularis and triangularis), and the left superior temporal gyrus (STG) and superior temporal sulcus (STS). These activations were largely contained within the language network (Figure 3). Right hemisphere regions homotopic to language areas were modulated by linguistic demand at the voxelwise threshold, but did not reach significance after correction for multiple comparisons. The regions that were negatively modulated by linguistic demand were bilateral and largely reflected the default mode network: the angular gyrus, precuneus, and posterior cingulate (Figure 2 and Table 2). In the left angular gyrus, this overlapped with the language network (Figure 3).

**Table 2.** Coordinates of activated regions.

**Brain region(s)**	**Extent (mm^3^)**	**Max *t***	**MNI coordinates**			***p***
			***x***	***y***	***z***	
*Linguistic demand*						
Left IFG, pars opercularis and triangularis	11,984	7.80	−45	21	10	0.0078
Left STG and STS	6,360	5.98	−57	−25	−1	0.036

*Negative linguistic demand*						
Bilateral angular gyri, precuneus, and posterior cingulate, and right fusiform gyrus	79,616	11.20	−1	−57	26	0.0011
Right precentral and postcentral gyri	6,840	6.03	41	−22	58	0.029
Left fusiform gyrus	5,680	10.31	−28	−38	−17	0.039

*Interaction of linguistic demand by modality*						
No activations						

*Negative interaction of linguistic demand by modality*						
Left inferior temporal gyrus, fusiform gyrus, and inferior occipital lobe	13,224	4.97	−41	−56	−15	0.020

*Perceptual demand*						
Right anterior insula	6,480	4.67	40	29	2	0.029

*Negative perceptual demand*						
Bilateral precuneus	17,112	4.72	2	−50	43	0.0056
Left angular gyrus	13,704	6.24	−47	−60	30	0.0067
Left superior frontal gyrus	11,232	5.25	−20	36	41	0.012
Left posterior insula	6,352	5.88	−41	−13	18	0.036

*Interaction of perceptual demand by modality*						
Left STG and MTG	15,544	4.53	−59	−26	1	0.026
Bilateral posterior cingulate and precuneus	14,416	4.46	1	−50	23	0.027
Bilateral ventromedial prefrontal cortex	12,472	6.67	−1	56	0	0.029
Right STG and MTG	10,808	4.47	60	−13	−8	0.033

*Negative interaction of perceptual demand by modality*						
Bilateral intraparietal sulcus, superior parietal lobule, occipito-temporal cortex, and cerebellum	134,032	7.83	−3	−70	5	0.0011
Left dorsal precentral gyrus and sulcus, left pre-supplementary motor area	15,888	5.26	−29	0	47	0.014
Right dorsal precentral gyrus and sulcus	7,096	5.67	25	−2	55	0.045

*Interaction of domain by difficulty*						
Left IFG, pars opercularis and triangularis, inferior frontal sulcus, ventral precentral gyrus, anterior STS and STG	22,040	8.76	−46	16	13	0.0033

*Negative interaction of domain by difficulty*						
No activations						

*Interaction of domain by difficulty by modality*						
Left intraparietal sulcus, superior parietal lobule, and occipito-temporal cortex	34,096	6.39	−32	−72	17	0.0022
Right intraparietal sulcus, superior parietal lobule, and occipito-temporal cortex	29,992	5.74	31	−70	20	0.0022

*Negative interaction of domain by difficulty by modality*						
Bilateral precuneus and posterior cingulate	19,096	5.56	1	−54	26	0.010
Right STG and MTG	9,904	5.29	60	−13	−10	0.031

*Note*. Coordinates are centers of mass. IFG = inferior frontal gyrus, STG = superior temporal gyrus, STS = superior temproal sulcus, MTG = middle temporal gyrus, MNI = Montreal Neurological Institute.

**Figure 4.**
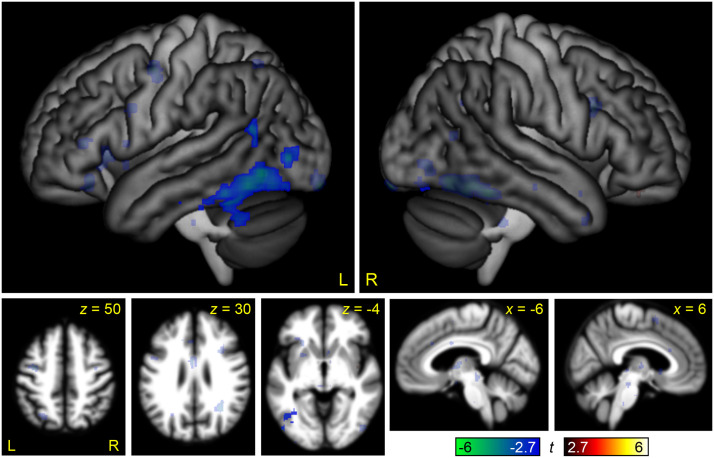
Brain regions differentially modulated by linguistic demand in the auditory and visual modalities. Regions that were more modulated by linguistic demand in the auditory modality than the visual modality would have been shown in hot colors (but there were none), while regions that were more modulated by linguistic demand in the visual modality than the auditory modality are shown in cool colors. Opaque = statistically significant, corrected for multiple comparisons; transparent = voxelwise *p* < 0.005, but did not meet cluster extent threshold.

This contrast can be compared to the parallel contrast in the visual modality in Quillen et al. (2021, Figure 2). To statistically compare these maps, the between-groups interaction of linguistic demand by modality (auditory, written) was computed (Figure 4 and Table 2). The frontal regions modulated by linguistic demand were similar across the auditory and written modalities. The left temporal regions that were significantly modulated in the auditory modality were modulated below threshold in the visual modality, but this difference was not significant in the interaction map. The most striking discrepancy was that there was no bilateral occipito-temporal modulation in the auditory modality; this difference between modalities was significant in the left hemisphere, as seen in the interaction map.

### Brain Regions Modulated by Non-Linguistic Demand

**Figure 5.**
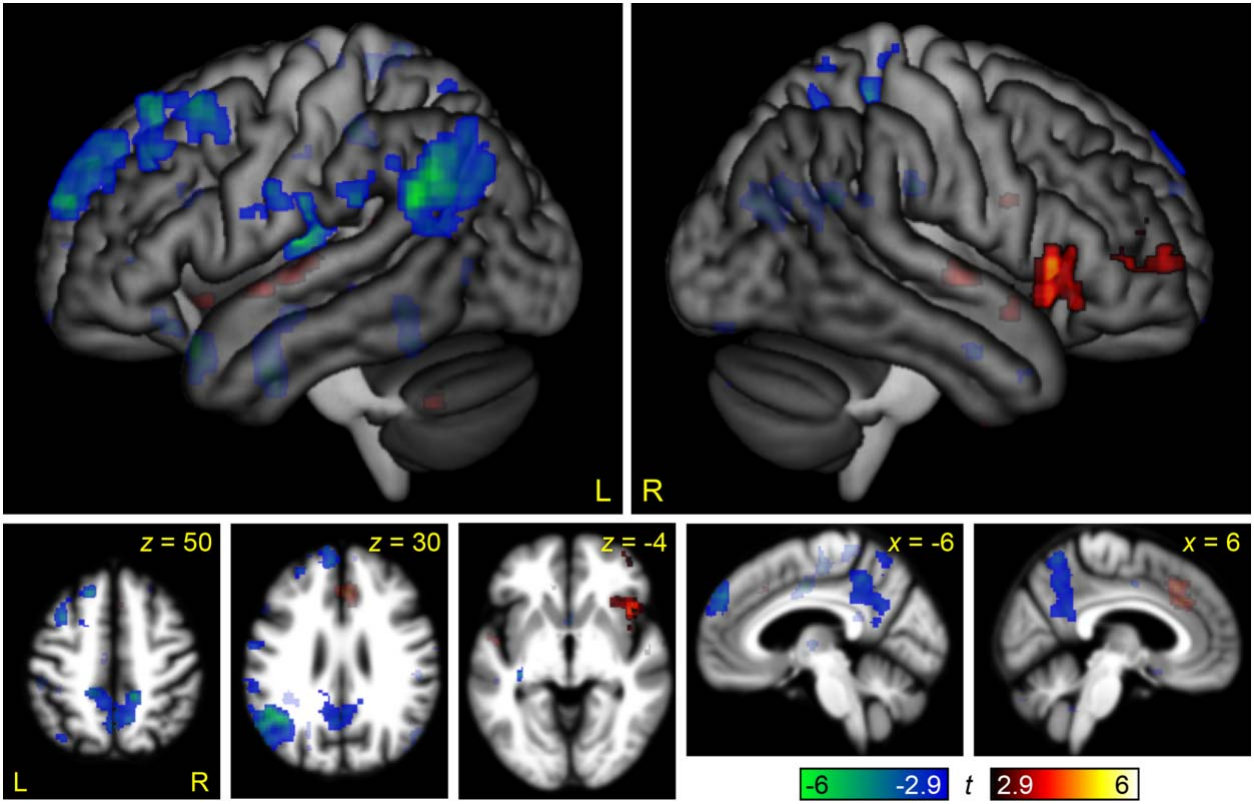
Brain regions modulated by non-linguistic demand. The contrast between the Melodic Difficult and Melodic Easy conditions is shown in hot colors, while the reverse contrast is shown in cool colors. Opaque = statistically significant, corrected for multiple comparisons; transparent = voxelwise *p* < 0.005, but did not meet cluster extent threshold.

The contrast between the Melodic Difficult and Melodic Easy conditions was used to identify brain regions modulated by non-linguistic demand in the auditory modality (Figure 5 and Table 2). The only region that was significantly modulated by non-linguistic demand was the right anterior insula. The bilateral auditory regions of the STG were also modulated at the voxelwise threshold, potentially reflecting the greater acoustic complexity of the Melodic Difficult condition, but this activation did not reach statistical significance after correction for multiple comparisons. Several default mode regions were deactivated.

**Figure 6.**
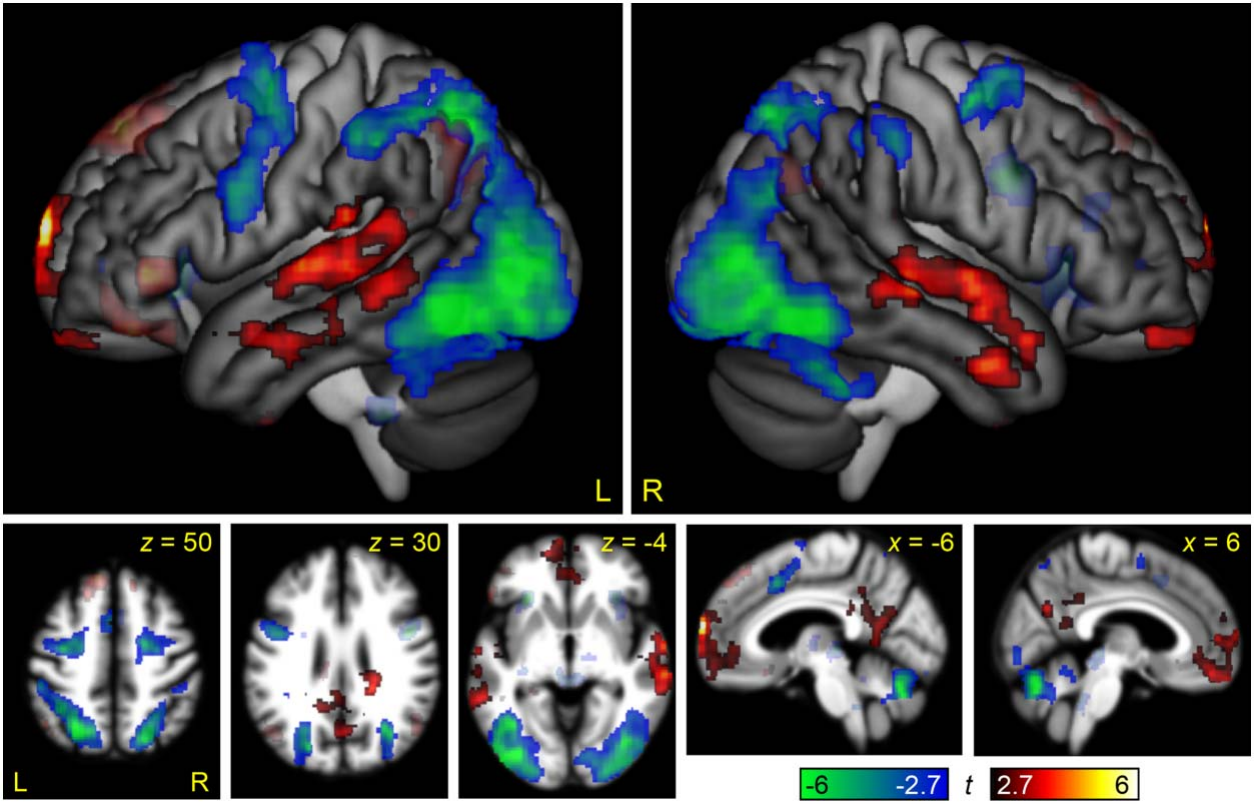
Brain regions differentially modulated by non-linguistic demand in the auditory and visual modalities. Regions that were more modulated by non-linguistic demand in the auditory modality than the visual modality are shown in hot colors, while regions that were more modulated by non-linguistic demand in the visual modality than the auditory modality are shown in cool colors. Opaque = statistically significant, corrected for multiple comparisons; transparent = voxelwise *p* < 0.005, but did not meet cluster extent threshold.

This contrast can be compared to the parallel contrast in the visual modality in Quillen et al. (2021, Figure 4). To statistically compare these maps, the between-groups interaction of non-linguistic demand by modality (auditory, written) was computed (Figure 6 and Table 2). This analysis revealed a positive interaction in the bilateral STG: As just noted, these regions were (non-significantly) modulated by non-linguistic demand in the auditory modality but were significantly deactivated by non-linguistic demand in the visual modality. There was a negative interaction throughout much of the MD network, reflecting the fact that the MD network was strongly modulated by non-linguistic demand in the written modality, but not in the auditory modality, except for the right anterior insula.

### Interaction of Domain by Difficulty

**Figure 7.**
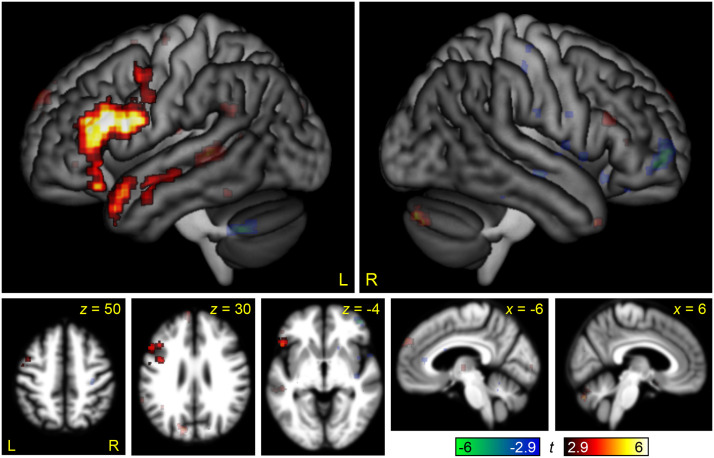
The interaction between linguistic demand and non-linguistic demand. Regions where modulation by linguistic demand was greater than modulation by non-linguistic demand are shown in hot colors, while the reverse contrast is shown in cool colors. Opaque = statistically significant, corrected for multiple comparisons; transparent = voxelwise *p* < 0.005, but did not meet cluster extent threshold.

To directly compare modulation by linguistic and non-linguistic demand in the auditory modality, we computed a whole brain interaction contrast of domain (linguistic, non-linguistic) by difficulty (Figure 7 and Table 2). This interaction map showed that the left IFG and left anterior STS and STG were differentially modulated by linguistic demand, while there were no regions that were differentially modulated by non-linguistic demand.

**Figure 8.**
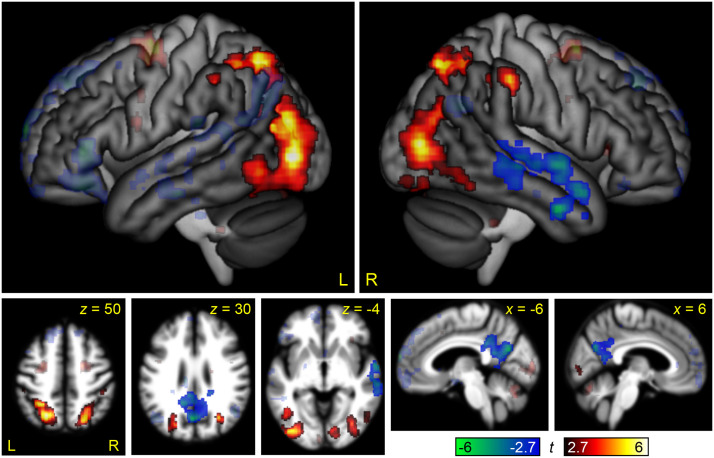
The three-way interaction between domain (linguistic, non-linguistic), modality (auditory, visual), and difficulty. Regions where modulation by linguistic demand was greater than modulation by non-linguistic demand in the auditory modality relative to the visual modality (or equivalently, modulation by non-linguistic demand was greater than modulation by linguistic demand in the visual modality relative to the auditory modality) are shown in hot colors, while the reverse contrast is shown in cool colors. Opaque = statistically significant, corrected for multiple comparisons; transparent = voxelwise *p* < 0.005, but did not meet cluster extent threshold.

This contrast can be compared to the parallel interaction contrast in the visual modality in Quillen et al. (2021, Figure 6). To statistically compare these maps, the three-way between-groups interaction of domain by difficulty by modality was computed (Figure 8 and Table 2). This map showed a positive three-way interaction in much of the posterior MD network, reflecting its differential modulation by non-linguistic relative to linguistic demand in the visual but not the auditory modality, and a negative three-way interaction in the right STG and middle temporal gyrus, driven by strong negative modulation of this region by non-linguistic demand in the visual modality only.

### Functionally Defined Regions of Interest

**Figure 9.**
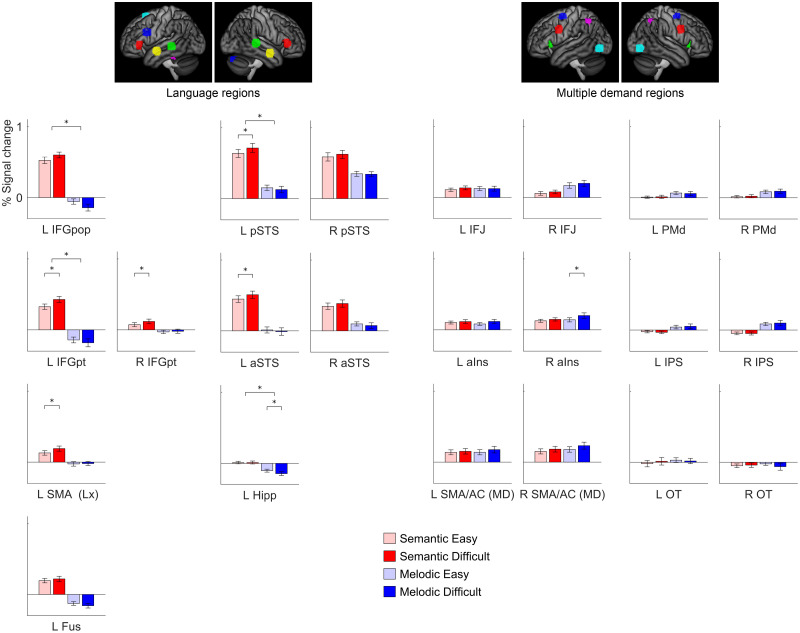
Functional region of interest analysis. Signal change for the four conditions in language regions, right hemisphere regions homotopic to language regions, and multiple demand regions. Error bars show standard error of the mean. L = left; R = right; IFGpop = inferior frontal gyrus, pars opercularis; IFGpt = inferior frontal gyrus, pars triangularis; SMA (Lx) = pre-supplementary motor area identified with language contrast, centered on –6, 16, 58; Fus = fusiform gyrus; pSTS = posterior superior temporal sulcus; aSTS = anterior superior temporal suclus; Hipp = hippocampus; IFJ = inferior frontal junction; aIns = anterior insula; SMA/AC (MD) = pre-supplementary motor area and anterior cingulate identified with MD contrast, centered on ±6, 16, 46; PMd = dorsal premotor cortex; IPS = intraparietal sulcus; OT = occipito-temporal cortex.

A limitation of voxelwise group analyses is that participants are aligned anatomically but not functionally, potentially resulting in the conflation of adjacent but functionally distinct brain regions (Blank et al., 2017; Fedorenko et al., 2010). Therefore, we next carried out a ROI analysis in which we plotted signal change as a function of domain (linguistic, non-linguistic) and difficulty (easy, difficult) in language network and MD network nodes that were functionally defined in individual participants (Figure 9 and Supplementary Table 2).

In the language network, several regions were significantly modulated by linguistic demand: the left IFG pars triangularis, left SMA/anterior cingulate, left posterior STS, left anterior STS, and right IFG pars triangularis, generally mirroring the findings from the whole brain analysis. No regions were modulated by non-linguistic demand (except that the left hippocampus was negatively modulated). There were significant domain by difficulty interactions in a number of regions. The broad patterns in the language network were quite similar to what was observed in the visual modality; compare Quillen et al. (2021, Figure 7), but note that that figure is arranged differently. Statistical comparisons between the present data set (auditory modality) and Quillen et al.’s data (visual modality; Supplementary Table 3) showed that for linguistic demand, only the left fusiform gyrus differed by modality, being more modulated by linguistic demand in the visual modality. For non-linguistic demand, the left fusiform gyrus was more modulated by non-linguistic demand in the visual modality, while the left posterior STS showed a larger negative modulation in the visual modality.

In the MD network, there was almost no modulation by either linguistic demand or non-linguistic demand, the sole exception being the right anterior insula, which was modulated by difficulty in the non-linguistic domain, as also seen in the whole brain analysis. There were no interactions of domain by difficulty. These findings contrast sharply with what was observed in the visual modality (Quillen et al., 2021, Figure 7), where every MD region was modulated by both linguistic and non-linguistic demand, the extent of modulation always greater for non-linguistic demand. Statistical comparisons between the present data set (auditory modality) and Quillen et al.’s data (visual modality; Supplementary Table 3) showed that 7 of 12 MD regions were less modulated by linguistic demand in the auditory modality, and all 12 MD regions were less modulated by non-linguistic demand in the auditory modality.

## DISCUSSION

We hypothesized that the modality of stimulus presentation—auditory or visual—would have minimal effect on the brain regions modulated by linguistic and non-linguistic demand. Within the language network, this hypothesis was largely borne out: Language regions were modulated by linguistic demand and not by non-linguistic demand in the auditory modality, similar to what was previously observed in the visual modality (Quillen et al., 2021). However, in the MD network, our hypothesis was clearly disconfirmed: MD regions were generally modulated neither by linguistic demand nor non-linguistic demand in the auditory modality, in striking contrast to what was previously observed in the visual modality (Quillen et al., 2021) and in many previous manipulations of cognitive demand (Duncan & Owen, 2000; Fedorenko et al., 2013).

### Modulation of the Language Network by Linguistic Demand

The core left hemisphere language regions in the IFG and STS were modulated by linguistic demand, as revealed by the whole brain analysis (Figure 2 and Figure 3) and the ROI analysis (Figure 9). The right IFG pars triangularis was also somewhat modulated, reaching significance in the ROI analysis (Figure 9). None of these regions were modulated by non-linguistic demand. The modulation of language regions by linguistic demand, or variables such as syntactic complexity, word frequency, or ambiguity, has been reported in many previous studies (Binder et al., 2005; Bornkessel et al., 2005; Graves et al., 2007, 2010; Just et al., 1996; Makuuchi et al., 2009; Obleser et al., 2011; Quillen et al., 2021; Rodd et al., 2005; Roskies et al., 2001; Sabsevitz et al., 2005; Shain et al., 2020, 2022; Stromswold et al., 1996; Thompson et al., 2007; Wehbe et al., 2021; Wilson et al., 2016).

The modulation of language regions by linguistic demand was broadly similar to what was previously observed in the analogous study of Quillen et al. (2021) in the visual modality. One apparent difference was that the core left temporal language region in the STS was modulated by linguistic demand in the auditory modality, while in the visual modality, this region was statistically significant only in the interaction analysis, where negative modulation by non-linguistic demand contributed to a significant effect. However, this difference between modalities was not significant in either the whole brain or ROI analyses between groups. The only significant difference between groups was observed in the left fusiform gyrus, which was modulated by linguistic demand in the written modality but not the auditory modality, consistent with the role of this region in processing orthographic representations (e.g., Vinckier et al., 2007).

Although the regions that were modulated by linguistic demand were mostly language regions (i.e., activated for the semantic conditions relative to the melodic conditions), the converse was not true. In particular, the left angular gyrus was identified as a language region, yet was actually negatively modulated by linguistic demand (Figure 3). As the language network shades into the default mode network in the angular gyrus, it may be that the more challenging overt semantic processing engendered by the Semantic Difficult condition cannot counteract the suppression of endogenous conceptual processing associated with performance of an attention-demanding task (Binder et al., 1999; McKiernan et al., 2003; Quillen et al., 2021).

### Modulation of the MD Network by Linguistic Demand

We observed no modulation of the MD network by linguistic demand in either whole brain analyses (Figure 2) or ROI analyses (Figure 9). This contrasts strikingly with the findings of Quillen et al. (2021), where the analogous experiment in the visual modality revealed modulation of all MD nodes by linguistic demand, albeit to a lesser extent than non-linguistic demand. The lack of modulation of MD regions by linguistic demand accords with several recent studies of auditory narrative comprehension that have reported that the MD network is not modulated by surprisal (Shain et al., 2020), online measures of incremental processing load (Wehbe et al., 2021), or working memory for sentence processing (Shain et al., 2022).

It is not clear why the linguistic demand contrast activated the MD network in the visual modality but not in the auditory modality. The visual and auditory semantic decision tasks used in the two studies involved assessment and comparison of the meanings of the two words, not their visual or auditory forms. The effects of task difficulty on accuracy and reaction time were very similar across the two modalities. Some MD nodes, in particular the dorsal premotor and intraparietal sulcus nodes, belong to the dorsal attention network and are involved in spatial attention and eye movements (Corbetta & Shulman, 2002, 2011); however, it is not obvious that the Semantic Difficult condition makes greater visual demands than the Semantic Easy condition. Whatever the reason might be, the present findings make clear that linguistic demand does not inherently modulate the MD network (Shain et al., 2020, 2022; Wehbe et al., 2021). The modulation of the MD network by linguistic demand in Quillen et al. (2021) must reflect one or more aspects of the task other than the semantic decision itself, since the same semantic decision did not modulate the MD network in the present study.

Perhaps even more surprisingly, we also observed almost no modulation of the MD network by non-linguistic demand. The only MD node that was modulated was the right anterior insula. This finding contrasts sharply with the analogous experiment in the visual modality, in which non-linguistic demand robustly modulated all nodes of the MD network (Quillen et al., 2021), and also with a recent study of working memory in the visual and auditory modalities, in which the contrasts between 3-back and 1-back conditions revealed almost identical maps in the two modalities (Assem et al., 2022).

We do not know why the non-linguistic demand contrast did not modulate the MD network. Structurally, the melodic matching task is quite similar to the perceptual matching task used in Quillen et al. (2021). Both involve two strings of elements, in which mismatch trials involve a single element differing between the two strings. Our accuracy and reaction time data confirmed that the 5-tone melodies were much more difficult than the 2-tone melodies, as intended. All participants performed above chance on both conditions, indicating that they did not simply give up on the difficult condition. While reaction times did not differ as much between the two conditions as in Quillen et al. (2021), it is notable that reaction times differed even less between the easy and difficult conditions in Assem et al. (2022), in which the MD network was robustly modulated by difficulty, so this cannot be the whole explanation. Whatever the reason may be, we can infer that not all cognitively demanding tasks differentially engage the MD network as a function of difficulty. It is clear that most tasks do (Fedorenko et al., 2013), but our data suggest that not all tasks do. A goal for future research will be to determine under precisely which circumstances the MD network is modulated by task difficulty.

### Limitations

Our study has several noteworthy limitations. First, although we manipulated the modality of presentation relative to Quillen et al. (2021), each study only investigated one type of linguistic task and one type of non-linguistic task. Second, the linguistic demand contrast also entails a difference in the extent of conceptual processing required between the easy and difficult conditions. Third, there are structural differences between the linguistic and non-linguistic conditions, such that the former involves a search for connections, leading to a button press, while the latter involves a search for differences, leading to withholding of a button press. Each of these limitations also applies to the study of Quillen et al. (2021), where they are discussed in more detail.

A limitation specific to the present study is that the behavioral data revealed interactions of domain by difficulty for accuracy and reaction time. Therefore, the difficulty modulation was not perfectly matched across domains. However, the accuracy and reaction time interactions were in different directions, suggesting that it was not the case that the magnitude of the difficulty manipulations differed across domains. Another related issue is that the effects of the difficulty manipulations on accuracy and reaction time differed somewhat between the present study and the previous study in the visual modality (Quillen et al., 2021). This is a limitation to interpretation of the between-groups analyses that we ran, because we cannot entirely rule out that aspects of the group differences reflected differences in the effectiveness of the difficulty manipulations, rather than the difference in modalities. We believe this is only a minor limitation, because the behavioral effects of the difficulty manipulations (on accuracy and reaction time) in the two studies were ultimately quite similar, whereas some of the differences in activation patterns between groups were dramatic, implying that it was most likely the modality difference (visual vs. auditory) that was responsible for the findings. It would be worthwhile to confirm our findings in future work using a fully within-subjects design with the behavioral effects of the difficulty manipulation more precisely matched across domains and modalities.

### Implications for Studies of Language Processing in Clinical Populations

The motivation for this work was to better understand the neural correlates of linguistic demand, because it is so frequently a confound in clinical studies (Geranmayeh et al., 2014; Quillen et al., 2021; Wilson & Schneck, 2021). This may include comparisons between individuals with aphasia and matched controls, between patients differing in the nature or severity of their aphasia, between time points in patients recovering or declining over time, and so on. Developmental studies face similar challenges, in that it can be difficult to disentangle changes in organization of the language network from changes in performance due to development (Olulade et al., 2020). Several studies have attempted to vary task demands between groups in order to ameliorate this problem (Berl et al., 2010, 2014; Brownsett et al., 2014; Gaillard et al., 2007; Heiss et al., 1999; Raboyeau et al., 2008; Sharp et al., 2004, 2010; Wilson et al., 2018, 2019; You et al., 2011), but it has proven challenging to completely account for differences in language processing abilities (Wilson & Schneck, 2021).

One conclusion that can clearly be drawn is that core left hemisphere frontal and temporal language regions are modulated by linguistic demand. This means that increased signal in these regions in individuals for whom language tasks are more difficult needs to be interpreted carefully, because it may reflect task demands. The right IFG, pars triangularis, homotopic to the left frontal language area, is also somewhat modulated by linguistic demand, potentially accounting for its frequently reported involvement in language processing in post-stroke aphasia (Wilson & Schneck, 2021).

The situation is much less clear for the MD network, which was not modulated by linguistic demand in the present study in the auditory modality, but was modulated in Quillen et al.’s (2021) analogous study in the visual modality. Linguistic demand in naturalistic contrasts, at least in the auditory modality, does not appear to modulate the MD network (Shain et al., 2020, 2022; Wehbe et al., 2021), so any modulation of the MD network by language tasks presumably reflects task effects. There are still good reasons to use explicit tasks in functional imaging studies of language in clinical populations, but our findings suggest that investigators interested in using language tasks to study language processing in clinical populations should empirically determine whether or not the difficulty of the planned task(s) modulates the MD network, so that results can be interpreted accordingly. It is also possible that individuals with language impairments may make differential demands on the MD network as a genuine compensatory strategy (Brownsett et al., 2014; Geranmayeh et al., 2017), and a more thorough understanding of how the MD network responds to different kinds of demand in different contexts will be important to further explore that intriguing hypothesis.

## ACKNOWLEDGMENTS

The authors thank Jillian Entrup and the VUIIS MRI technologists for assistance with performing the study, Ian Quillen and Melodie Yen for sharing data, Cory Shain, Ev Fedorenko, Jeff Binder, Ram Ramachandran, Michael de Riesthal, Yev Diachek, and Melissa Duff for valuable discussions and feedback, and all of the individuals who participated in the study.

## FUNDING INFORMATION

Stephen M. Wilson, National Institute on Deafness and Other Communication Disorders (https://dx.doi.org/10.13039/100000055), Award ID: R01 DC013270.

## AUTHOR CONTRIBUTIONS

**Mackenzie Philips**: Formal analysis; Investigation; Visualization; Writing – original draft; Writing – review & editing. **Sarah M. Schneck**: Conceptualization; Investigation; Writing – review & editing. **Deborah F. Levy**: Formal analysis; Investigation; Visualization; Writing – review & editing. **Stephen M. Wilson**: Conceptualization; Formal analysis; Funding acquisition; Methodology; Software; Visualization; Writing – original draft; Writing – review & editing.

## DATA AVAILABILITY STATEMENT

The underlying data for the key analyses described are available at: https://doi.org/10.17605/OSF.IO/MJ4EZ.
