# Supplementary material for: Modality-Specificity of the Neural Correlates of Linguistic and Non-Linguistic Demand
Source: Neurobiol Lang (Camb). 2023 Sep 18;4(4):516–35. doi: 10.1162/nol_a_00114 (PMC10575553; doi:10.1162/nol_a_00114)
Supplement: Supplementary file 1 [file nol-4-4-516-s001.pdf]

Supplementary material for Philips et al., ‘Modality-specificity of the neural correlates of linguistic and non-linguistic demand’

Supplementary Table 1 Centroids for regions of interest

Region of interest	Centroid		
	x	*y*	*z*
Language network			
L IFGpop	-46	14	26
L IFGpt	-48	28	0
R IFGpt	52	30	4
L SMA (Lx)	-6	16	58
L Fus	-38	-38	-22
L pSTS	-56	-36	-2
R pSTS	54	-32	4
L aSTS	-58	-6	-12
R aSTS	56	-4	-16
L Hipp	-26	-14	-16
MD network			
L IFJ	-46	4	32
R IFJ	46	4	32
L aIns	-30	22	4
R aIns	30	22	4
L SMA/AC (MD)	-6	16	46
R SMA/AC (MD)	6	16	46
L PMd	-26	-4	56
R PMd	26	-4	56
L IPS	-30	-54	46
R IPS	30	-54	46
L OT	-34	-78	-6
R OT	34	-78	-6

Supplementary Table 2 Region of interest analysis for the auditory modality

Region of interest	Linguistic demand (*p*)	Non-linguistic demand (*p*)	Interaction (*p*)
Language network			
L IFGpop	.062	.055	.0001 *
L IFGpt	.0003 *	.33	< .0001 *
R IFGpt	.036 *	1	.14
L SMA (Lx)	.017 *	1	.093
L Fus	.46	.16	.075
L pSTS	.0097 *	.76	.025 *
R pSTS	.10	1	.34
L aSTS	.019 *	.94	.068
R aSTS	.13	.83	.13
L Hipp	1	.0010 †	.036 *
MD network			
L IFJ	.37	1	.75
R IFJ	.76	.47	1
L aIns	.98	.25	.97
R aIns	.72	.033 *	.66
L SMA/AC (MD)	.99	.49	.99
R SMA/AC (MD)	.45	.097	1
L PMd	1	1	.98
R PMd	1	1	1
L IPS	.99	1	.96
R IPS	1	1	1
L OT	.59	1	.98
R OT	1	.93	.97

P values for the effects of linguistic demand, non-linguistic demand, and the interaction of domain by difficulty, in the language network and the MD network. All *p* values were corrected for multiple comparisons by permutation testing. * = $p < .05$; † = negative modulation, $p < .05$.

Supplementary Table 3 Region of interest analysis for the between-groups comparison of the auditory and visual modalities

Region of interest	Linguistic demand (*p*)	Non-linguistic demand (*p*)
Language network		
L IFGpop	.25	.094
L IFGpt	.38	.17
R IFGpt	.98	.94
L SMA (Lx)	1	.98
L Fus	.022 †	.025 †
L pSTS	1	.049 *
R pSTS	1	.19
L aSTS	1	.095
R aSTS	1	.10
L Hipp	.75	.96
MD network		
L IFJ	.0025 †	< .0001 †
R IFJ	.042 †	< .0001 †
L aIns	.19	.0018 †
R aIns	.084	< .0001 †
L SMA/AC (MD)	.035 †	< .0001 †
R SMA/AC (MD)	.024 †	< .0001 †
L PMd	.38	< .0001 †
R PMd	.11	< .0001 †
L IPS	.0008 †	< .0001 †
R IPS	.14	< .0001 †
L OT	.0071 †	< .0001 †
R OT	.017 †	< .0001 †

P values for the effects of linguistic demand and non-linguistic demand in the language network and the MD network. All *p* values were corrected for multiple comparisons by permutation testing. * = modulated more in the auditory modality, $p < .05$; † = modulated more in the visual modality, $p < .05$.
